# Modeling recurrent heart failure risk in type 2 diabetes: impact of flexible HbA1c trajectories using nonhomogeneous Poisson processes

**DOI:** 10.3389/fendo.2025.1472846

**Published:** 2025-04-21

**Authors:** Di Cui, Haiyan Xu, Xiuju Fu, Stefan Ma, Yong Mong Bee

**Affiliations:** ^1^ Institute of High Performance Computing (IHPC), Agency for Science, Technology and Research (ASTAR), Singapore, Singapore; ^2^ Public Health Group, Ministry of Health Singapore, Singapore, Singapore; ^3^ Department of Endocrinology, Singapore General Hospital, Singapore, Singapore

**Keywords:** type 2 diabetes, HbA1c trajectories, recurrent heart failure, glycemic control, SGLT2 Inhibitors, longitudinal data analysis, nonhomogeneous Poisson process, latent class growth models

## Abstract

**Background:**

Many clinical trials yielded inconsistent results regarding the effect of intensive glycated hemoglobin control on cardiovascular diseases in type 2 diabetes. We identified distinct HbA1c trajectories and their association with the recurrent hospitalization of heart failures (HHF) for patients with type 2 diabetes starting from the date of diabetes diagnosis.

**Methods:**

In this study, we included 194,258 patients who entered the SingHealth Diabetes Registry from 2013 to 2020. Their diagnoses of type 2 diabetes spanned the years 1960-2020, encompassing HbA1c measurements, records of HHF, and other cardiovascular complications. Latent class growth models (LCGM) with splines were used to extract the subgroups with distinct HbA1c trajectories. The association between HbA1c trajectories and the recurrent risk of HHF was investigated by nonhomogeneous Poisson processes (NHPP).

**Results:**

Eight distinct HbA1c trajectories were identified as follows: low stable (LowS, 22.2%), moderate low ascending (ModLowA, 12.7%), moderate high ascending (ModHighA, 11.5%), moderate low descending (ModLowD, 17.2%), moderate high descending (ModHighD, 10.1%), moderate high volatility (ModHighV, 10.1%), high with a sharp decline (HighSD, 8.0%), and high volatility (HighV, 10.2%). Using the Class LowS as a reference, the hazard ratios for recurrent HHF for the other classes are as follows: 0.79 for ModLowA, 1.30 for ModHighA, 1.17 for ModLowD, 1.89 for ModHighD, 1.94 for ModHighV, 1.25 for HighSD, and 2.88 for HighV. Considering recurrent HHFs, our NHPP model demonstrated predictive capability for type 2 diabetes patients’ future HHF events.

**Conclusions:**

Low baseline HbA1c levels are associated with a lower risk of recurrent HHF, while poor glycemic control significantly increases this risk. Our application of LCGM with splines effectively captures flexible, long-term HbA1c trajectories, while the innovative use of the NHPP model allows for precise modeling of HHF recurrence risk. This approach provides a foundation for personalized risk predictions and future HF management by incorporating dynamically updated risk factors.

## 1 Introduction

Individuals living with type 2 diabetes mellitus are widely considered to face an increased risk of heart failure (1, 2), a critical concern in healthcare. Type 2 diabetes is characterized by abnormal glucose homeostasis (3), with the degree of hyperglycemia typically measured using glycated hemoglobin (HbA1c), a marker associated with the risk of cardiovascular events (4, 5). Common oral antidiabetic medication such as SGLT2 inhibitors (SGLT2i) and sulfonylureas (SULP) have been observed to exert both positive and negative effects on reducing the risk of heart failure (6, 7), respectively, thereby introducing potential interference in the study of the relationship between HbA1c levels and heart failure risk.

Many clinical trials have yielded inconsistent results regarding the effect of intensive glycated hemoglobin control on cardiovascular diseases in type 2 diabetes (8–11). For example, a meta-analysis of prospective studies (8) found that chronic hyperglycemia is associated with an increased risk for cardiovascular outcomes, whereas a separate secondary analysis of 14,656 patients with type 2 diabetes (10) identified a U-shaped relationship with nadir around 7%. In light of these discrepancies, recent research highlights the necessity for individualized medical treatment of type 2 diabetes to account for the heterogeneity within the patient population (12, 13). Furthermore, it is essential to recognize the significance of HbA1c variability as a risk factor for cardiovascular complications (14, 15).

In addressing these challenges, latent class growth model (LCGM) is an efficient tool that enables the simultaneously identifying the subgroups of patients with distinct HbA1c trajectories and capturing HbA1c’s variation over time. By stratifying the population into homogeneous subgroups and considering the repeated measurements of HbA1c, the model enhances the predictive power for individual risk assessment and intervention (16, 17). Previous research has classified patients using LCGM (18–20), considering fixed-time HbA1c measurements (21, 22).

The prevailing approach in modeling HbA1c trends relies on parametric forms in most existing studies. For example (23), considered linear trends, while our prior research (24) employed a logarithmic function, which demonstrated optimality among several candidate parametric models. In contrast, a nonparametric model exhibits flexibility in capturing HbA1c trajectories, offering a more accurate representation of real-world variations. Notably, our current study expands upon our previous work by incorporating patients diagnosed with type 2 diabetes before the initiation of HbA1c records, extending the diagnosis year from 2013 to 1960. This expansion enriches our cohort, yielding a more comprehensive cohort for study.

Our prior research (24) has proved the predictive ability of latent classes on hospitalization of heart failure (HHF), which solely focused on the initial event following type 2 diabetes diagnosis. However, cardiovascular events usually occur more than once, and for patients diagnosed early, the first HHF post-diagnosis may be missing. Therefore, our current work models the recurrent processes of HHF starting from when covariate information becomes available, addressing the data limitations and enhancing our ability to predict future HHFs.

Commencing from the initiation of type 2 diabetes, this study advances existing research on individualized HbA1c trajectories by introducing three key innovative contributions. First, employing the latent class growth model with B-splines trajectories enables identifying the potential subgroups among HbA1c trajectories over an extended period – specifically, spanning 62 years from the date of type 2 diabetes diagnosis – for a significant cohort of 194,258 patients, each with HbA1c measurements recorded from 1998 or the time of type 2 diabetes diagnosis (whichever is later) to 2021 or the time of the patients’ departure from the system (whichever is earlier). Second, the study delves into the clinical implications of these subgroups on the risk of recurrent HHF through a precise model – nonhomogeneous Poisson processes (NHPP) – that takes into account the recurrent nature of HHF events with incomplete records spanning from 2007 to 2020. Third, by incorporating the identified HbA1c subgroups, the NHPP enables personalized predictions of future heart failure occurrences. It facilitates the ongoing prediction of HHF risk by incorporating continuously updated risk factors, including monitoring the evolution and dynamics of patients’ HbA1c and other cardiovascular complications recorded from 2013 onwards.

## 2 Materials and methods

### 2.1 Data source and study population

The SingHealth Diabetes Registry (SDR) is a comprehensive database that collects the electronic medical records of type 2 diabetes patients visiting the healthcare institutions within the Singapore Health Services (SingHealth), one of three health clusters in Singapore (25). The SDR contains casemix variables including demographic factors, diagnosis profile, treatment factors and anthropometric variables, as well as outcome variables including laboratory results, clinical episodes, surgical procedures and vaccinations.

This study exclusively included type 2 diabetes patients who visited SingHealth from 2013 to 2020, totaling 194,265 subjects documented in the SDR with one or more HbA1c records. The diagnoses for these subjects spanned from 1955 to 2020, but only 7 subjects were diagnosed between 1955 and 1959, and they were thus removed to balance the study population distribution. HbA1c records are available for the period between 1998 and 2021. Records of HHF encompass the years 2007 to 2020, while records of other cardiovascular complications cover the years 2013 to 2020. To conduct a fair comparison across all subjects, the baseline of the study is designated as the date of diabetes diagnosis (DDD) (21, 24, 26) instead of the date of the first HbA1c recorded in the SDR. If the type 2 diabetes diagnosis year is consistent with the year of the first HbA1c record, the DDD will be assumed as the date of the first HbA1c record. For subjects whose years do not match, the DDD will be set as a date that is uniformly selected from the year of type 2 diabetes diagnosis. Of these, 87,202 (44.8%) subjects require random imputation. Dropping these subjects would result in losing nearly half the information compared to completing the DDD. The final sample for HbA1c trajectory analysis comprised 194,258 subjects, covering a span of 62 years from 1960 to 2021. And the sensitivity analysis indicated that the robustness of the imputation method.

### 2.2 Statistical methods

The latent class growth model (LCGM) is utilized to cluster the distinct HbA1c trajectories. Different from traditional parametric forms, we make no assumptions about the shapes of the latent trajectory subgroups. Each trajectory is described by an unknown smooth function, which is approximated using B-splines because of their good properties both in theoretical and computational aspects (27). In addition to random errors, a random intercept is also considered for each subgroup to account for the within-subject heterogeneity.

The basic functions of B-splines are determined by the degree, the number and location of interior knots. The cubic spline (degree of 3) is smooth enough to fit the trajectory. For knot placement, we consider two strategies as follows (28):

1. Equidistant: each interval between knots has the same width.
2. Equipotent: each interval between knots includes the same number of data points.

The number of interior knots, ranging from 5 to 40, was tested for each strategy.

The optimal number of latent classes is determined by Bayesian Information Criterion (BIC) (29) using a 10-fold cross-validation procedure (30). The process of interactively determining knot placement and the number of latent classes is described as follows:

1. Set the initial number of classes $K_{0}$ ;
2. Calculate BIC values based on different numbers of interior knots, 5i, i=1,⋯,8 , for the two strategies with fixed $K_{0}$ . Knots placement is the pair with smallest BIC value.
3. Calculate BIC values using a 10-fold cross-validation procedure for different numbers of classes K=1,⋯,10 with fixed knots placement. The number of classes $K_{1}$ is the one with smallest BIC value.
4. Repeat steps 2 and 3 until the optimal selection of the number of knots, knot placement and the number of classes $K_{1}$ becomes stable.

Nonhomogeneous Poisson processes (NHPPs) were used to model the recurrent HHFs, acknowledging that the records of HHF recurrence for each patient are only available within a specific period. Define T as the weeks elapsed from DDD and $T_{0}=0$ . The observed HHF sequence for a subject is $\left\{T_{i},\;i=1,\;2,\;\cdots \right\}$ . For NHPP, the transformed time difference between two successive events follows a standard exponential distribution, that is,


$$\text{Λ}\left(T_{i}\right)-\text{Λ}\left(T_{i-1}\right)\sim Exp\left(1\right)$$

where $\text{Λ}\left(t\right)=\int_{0}^{t}\lambda \left(u\right)du$ . If λ(t)≡λ , the NHPP (λ(·)) becomes the Homogeneous Poisson process, HPP (λ) . The nonnegative function λ(t) is called intensity function. Referring to (31), λ(t) adopts the power-law form, that is,


$$\lambda \left(t|\theta ,\beta ,\gamma \right)=\left(\frac{\beta }{\eta }\right){\left(\frac{t}{\eta }\right)}^{\beta -1}\text{exp}\left(x^{\text{T}}\gamma \right),$$

where x is the vector of adjusted risk factors, η is the scale parameter, β is the shape parameter and γ is the effect of covariates. All analyses were conducted using R version 3.6.0.

## 3 Results

### 3.1 Model selection

HbA1c values could be roughly classified as low, moderate, and high levels, leading us to initially set the number of classes at 3 . Given the number of classes, the model with 30 equipotently placed interior knots yielded the smallest BIC value (**Supplementary Table 1**). The analysis then proceeded with these 30 fixed interior knots while exploring class numbers from 1 to 10. Through 10-fold cross-validation, the optimal number of latent classes was identified as 8, based on the lowest average BIC values obtained from the cross-validation (**Supplementary Table 2**). Subsequent recalculations of BIC values for all configurations (varying numbers of knots and knot placement strategies) with 8 latent classes confirmed that the model with 30 equally spaced interior knots continued to exhibit the minimum BIC value, as detailed in **Supplementary Table 3**.

### 3.2 HbA1c trajectories

**Figure 1:**
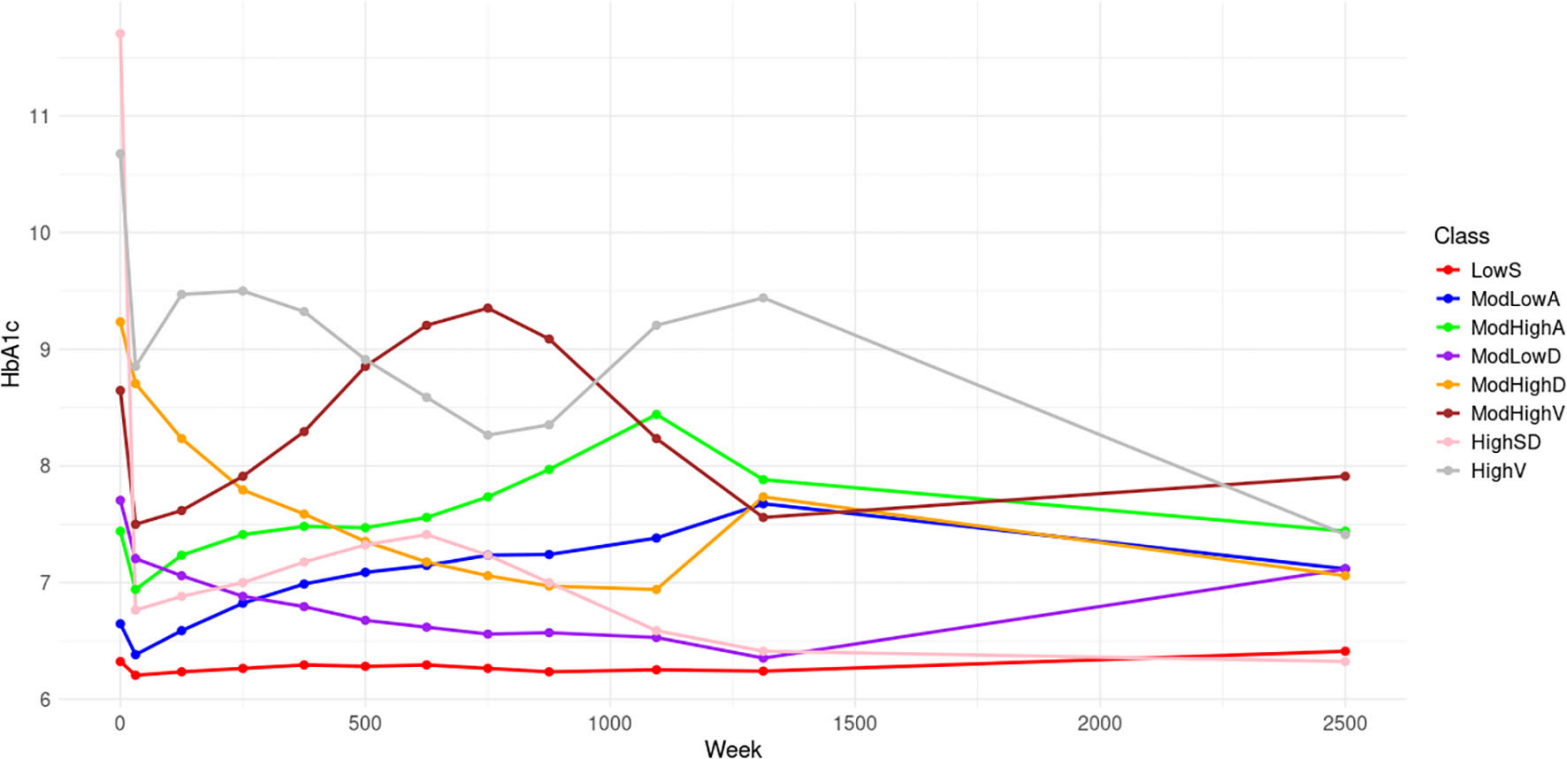
HbA1c trajectories of the 8 classes.

**Figure 1** displays the 8 trajectories approximated by B-splines with 30 equipotently placed interior knots. Low stable (LowS) comprising 22.2% of the population, has a baseline HbA1c level of 6.3% and maintains stable levels thereafter. Moderate low ascending (ModLowA), comprising 12.7% of the population, has a baseline HbA1c level of 6.7% and gradually ascends to 7.4% over 25 years. Moderate high ascending (ModHighA), comprising 11.5% of the population, has a moderately high starting HbA1c level of 7.4% and then shows ascending trend. Moderate low descending (ModLowD), comprising 17.2% of the population, has a baseline HbA1c level of 7.7% followed by descending trends over 25 years. The moderate high descending (ModHighD) trajectory makes up 10.1% of the population. It shows a descending trend with a lower HbA1c level than the baseline of 9.3%. Moderate high volatility (ModHighV), comprising 10.1% of the population, begins with a baseline HbA1c level of 8.6% followed by significant fluctuations. High with a sharp decline (HighSD) represents 8.0% of the population. These participants begin with the highest HbA1c values of 11.6% among all 8 classes, followed by a sharp drop to a near-normal HbA1c level. The high volatility (HighV) trajectory accounts for the remaining 10.2% of the population with a baseline HbA1c level of 10.8%, showcasing significant fluctuations.

Baseline characteristics of subjects by distinct eight HbA1c trajectories are presented in **Table 1**. The mean age varies across these classes, ranging from 50.2 years in the high fluctuation group to 63.7 years in the low stable group. Classes with elevated baseline HbA1c levels exhibit a greater proportion of male patients, spanning from 48.9% to 58.6%. The prevalence of non-Chinese patients is more pronounced in trajectories with higher HbA1c levels, ranging from 22.7% to 50.7%.

**Table 1:** The baseline characteristics of the 8 classes.

	LowS	ModLowA	ModhighA	ModLowD	ModHighD	ModHighV	HighSD	HighV
N (%)	43,170 (22.2)	24,702 (12.7)	22,262 (11.5)	33,358 (17.2)	19,635 (10.1)	15,766 (8.1)	15,516 (8.0)	19,849 (10.2)
Mean age at DDD	63.7 ± 12.2	58.3 ± 11.9	55.8 ± 13.0	59.4 ± 12.0	55.1 ± 12.4	51.7 ± 13.5	54.1 ± 12.0	50.2 ± 13.4
Male, %	48.9	50.8	52.2	50.6	52.3	53.8	55.2	58.6
Ethnicity, %								
Chinese	77.3	75.1	68.4	72.8	64.4	58.6	67.2	49.3
Malay	10.9	11.7	14.5	13.0	17.3	20.7	18.4	27.2
India	8.1	9.4	12.5	10.2	13.4	15.0	9.8	16.5
Others	3.7	3.8	4.6	4.0	4.9	5.7	4.6	7.0
HbA1c observed at DDD, %	72.8	68.7	69.8	71.6	65.1	63.1	74.4	67.6
Mean HbA1c at diagnosis of type 2 diabetes (SD)	6.27 (0.58)	6.70 (0.68)	7.41 (1.14)	7.73 (0.85)	9.28 (1.48)	8.63 (1.95)	11.60 (1.41)	10.81 (2.63)
Mean HbA1c Frequency (SD)	11.2 (12.4)	23.8 (19.8)	30.8 (24.7)	23.8 (23.1)	30.7 (25.9)	30.8 (24.3)	26.1 (23.4)	21.3 (20.8)
Mean observational period (SD), weeks	219.9 (236.2)	420.2 (300.9)	523.6 (327.5)	410.2 (345.5)	519.2 (355.3)	552.7 (313.5)	423.6 (336.0)	443.3 (319.5)
HHF observed, %	2.93	3.30	6.57	4.98	9.21	9.42	4.62	12.09
Established CVD, %	25.8	23.7	28.4	26.2	29.9	28.0	22.3	27.4
Prior IHD, %	16.9	15.9	18.9	17.2	19.1	18.3	13.5	17.3
Prior PAD, %	1.2	0.9	1.6	1.3	3.0	2.6	1.8	4.3
Prior HS, %	0.9	0.6	0.7	0.7	0.6	0.7	0.9	0.6
Prior IS, %	6.4	5.2	5.8	6.0	7.2	5.8	6.2	5.8
Prior TIA, %	1.6	1.3	1.2	1.2	1.1	1.1	1.0	0.9
Prior AF, %	4.7	3.4	3.8	4.0	4.0	3.5	2.7	3.3
Prior Neuropathy, %	1.0	0.7	1.3	1.3	2.2	2.3	1.9	4.0
Prior DPA, %	1.2	0.8	1.8	1.6	3.4	3.5	2.7	6.3
Prior Heart Failure, %	1.2	1.0	2.0	1.6	2.7	2.8	1.3	3.6
Acarbose, %	0.5	1.8	8.0	3.8	10.6	11.0	5.6	7.5
Metformin, %	30.1	52.1	68.2	66.5	73.5	73.4	80.8	73.7
Sulfonylureas, %	9.3	24.1	48.8	37.9	56.2	55.9	56.1	55.7
DPP-4i, %	2.8	4.3	9.6	6.2	11.4	13.0	10.6	12.0
SGLT2i, %	1.5	1.7	2.5	2.8	3.4	2.8	4.9	3.9

**Figure 2:**
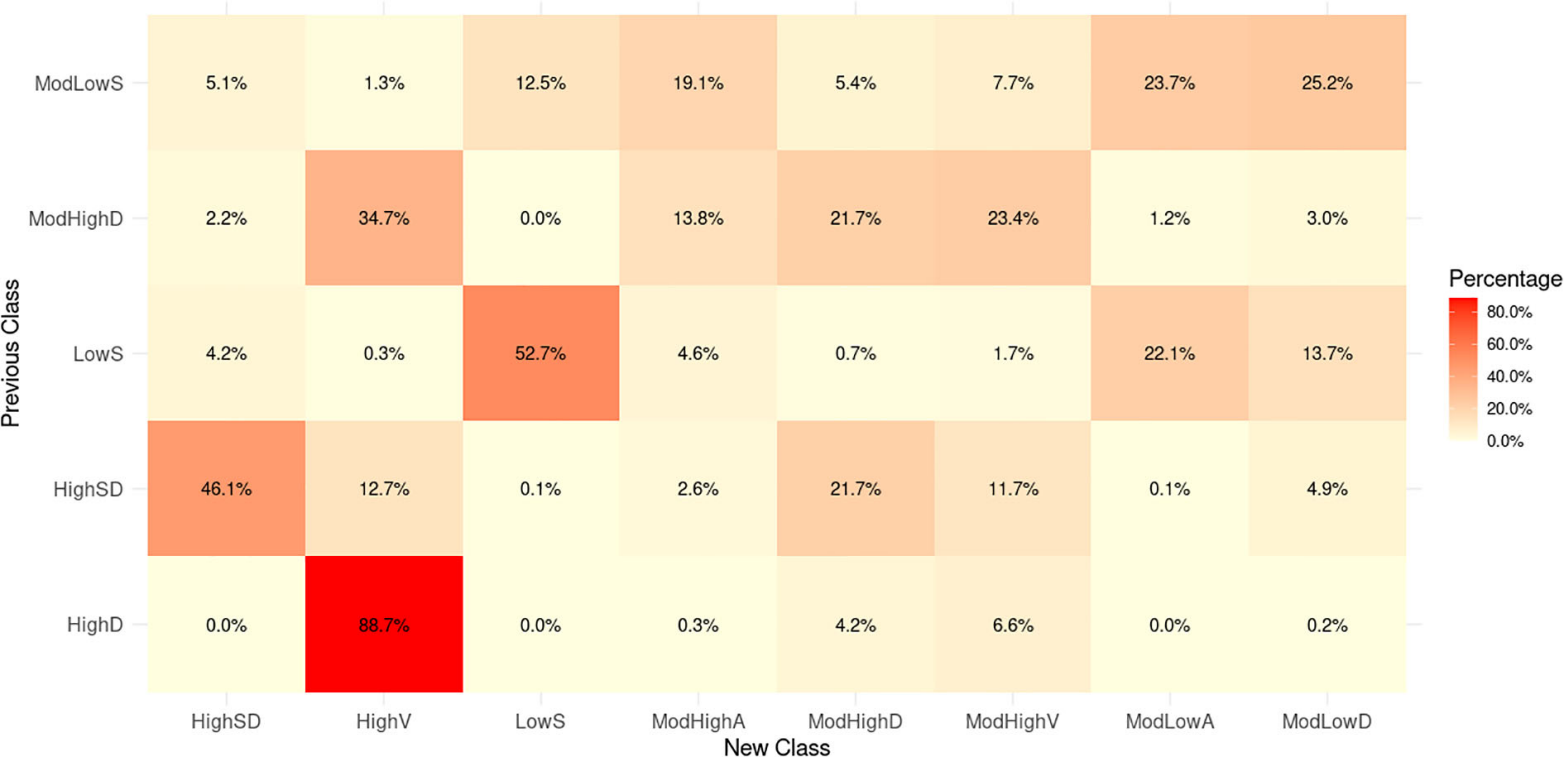
Heatmap of the coincidence rates.

We further compare our classification to our previous results of (24), which adopted a logarithmic LGCM based on the subset of the dataset used in this study. A total of 17,389 subjects were selected, each with at least 5 HbA1c samples across the span of at least 3 years from 2013 to 2019. Our previous work (24) classified the 17,389 subjects into five subgroups with: “low stable (35.5%, LowS), moderate low stable (41.1%, ModLowS), high descending (3.4%, HighD), high with a sharp decline (10.7%, HighSD) and moderate high descending (9.2%, ModHighD)”. We mapped the previous subgroups (classification derived in (24)) to the new subgroups (classification in this study). **Figure 2** shows that subjects in the previous Class LowS are mainly distributed in the new Class LowS, ModLowA, and ModLowD, all of which exhibit low HbA1c levels. For the previous Class ModLowS, the subjects appear evenly in the first four new classes with relatively low HbA1c levels. The coincidence rate between previous Class HighD and new Class HighV is up to 88.7%. The two classes both have highest HbA1c levels in their own population. The previous Class HighSD starts with a very high HbA1c level followed by a steep decline. New Class ModHighD and HighSD with high coincidence rates show a similar sharp downward trend. Subjects in the previous Class ModHighD are mainly concentrated in the new Class ModHighA, ModHighD, ModHighV, and HighV that also have high or moderately high HbA1c levels.

### 3.3 Association between HbA1c trajectories and risk of HHF

**Figure 3:**
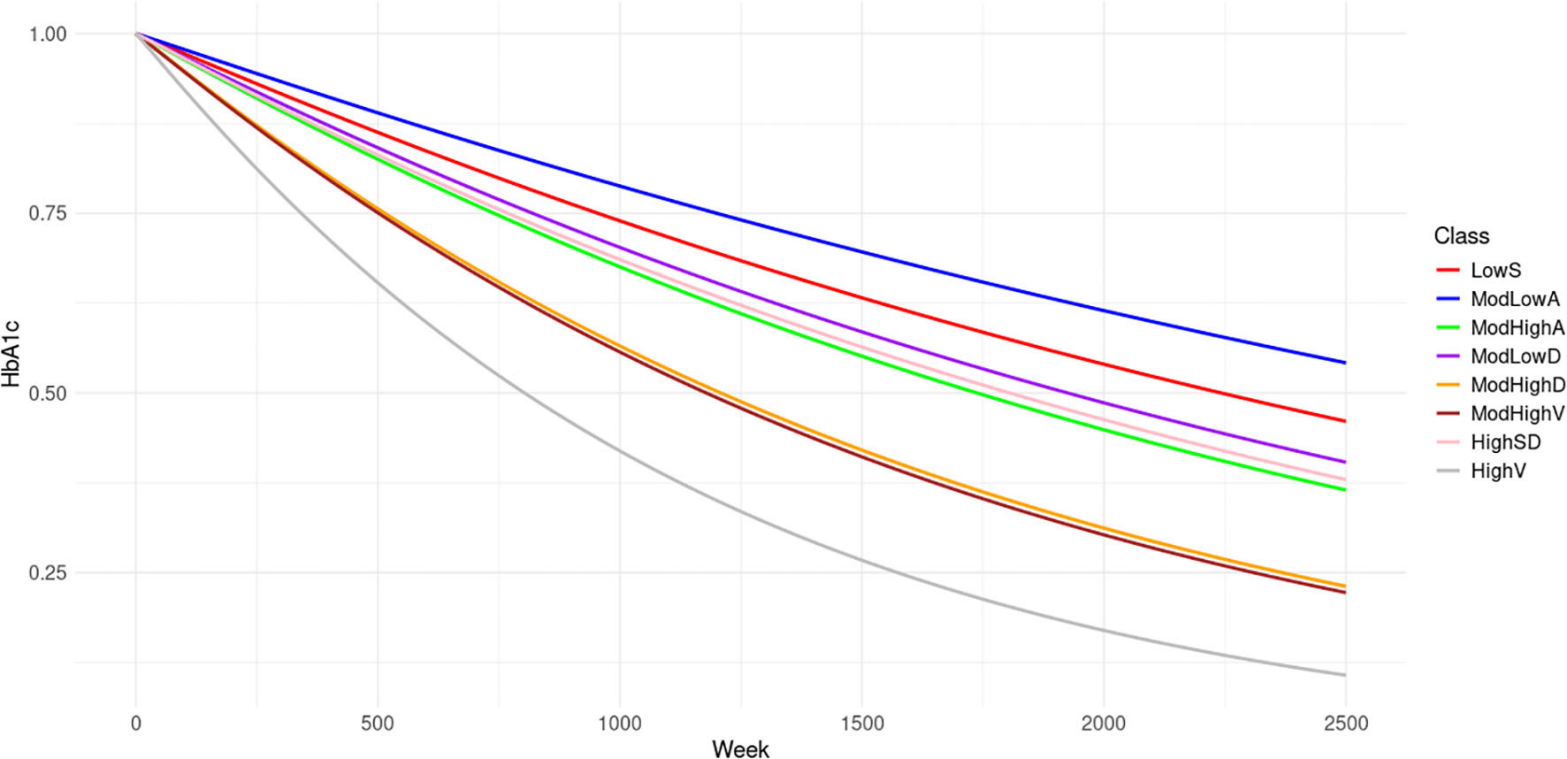
The mean survival probabilities of first HHF for the 8 classes.

Cardiovascular complication histories and records are only available from 2013 to 2020. To accurately obtain the effects of these complications on the risk of HHF, the earliest date of a patient having complete covariates is set as the truncated date. For each patient, if DDD is after the truncated date, the left bound of the observation window is 0. Otherwise, the left bound is the difference between the truncated date and DDD. Only the HHF records within the observation window are used to train the NHPP model. We first fit the NHPP model based on the eight latent classes distinguished by HbA1c trends. **Figure 3** displays the evolution of the mean survival probabilities for the first HHFs over time. The survival probabilities are highest in Class ModLowA. Classes LowS, ModLowD and HighSD share similar trends in survival probability, albeit slightly lower than those of Class ModLowA. In contrast, Classes ModHighA, ModHighD, ModHighV and HighV display considerably lower survival probabilities, with Classes ModHighD and ModHighV almost identical, and Class HighV exhibiting the lowest probability among them.

We further adjust the NHPP model with risk factors used in our prior research (24). **Table 2** summarizes hazard ratios and their significance after adjusting for population characteristics (age at diagnosis, ethnicity, gender, and baseline HbA1c), established cardiovascular disease (CVD), prior ischemic heart disease (IHD), prior peripheral arterial disease (PAD), prior ischemic stroke (IS), prior hemorrhagic stroke (HS), prior transient ischemic attack (TIA), prior atrial fibrillation (AF), prior neuropathy, prior diabetic peripheral angiopathy (DPA) and prior heart failure (HF). Prior complications here are defined as the medical history before the left bound of the observation window for each patient. The rate of patients with prior complications for each class is summarized in **Table 1**. Class ModLowA shows a significantly lower risk than Class LowS with hazard ratio HR = 0.79. Class ModHighA, ModHighD, ModHighV, and HighV exhibit significantly higher risk, as indicated by the hazard ratios HR = 1.30, 1.89, 1.94, and 2.88 when compared to Class LowS. The three classes share a common moderately high to high baseline HbA1c levels. Class HighSD, which has the highest baseline HbA1c level and decreases rapidly, eventually nearing the lower and stable levels observed in Class LowS, exhibits a significantly increased risk with an HR of 1.25. However, this risk is notably lower compared to classes ModHighD, ModHighV, and HighV. Among all the prior complications, prior CVD, prior IHD, prior HS, prior IS, prior TIA, prior AF, prior neuropathy, prior DPA and prior HF exhibit significant effects on the risk of HHFs. This study identifies four additional significant risk factors: prior IHD, prior HS, prior IS and prior TIA compared with our previous work. Both studies obtained the results that prior CVD and prior HHF have the greatest impact.

**Table 2:** Effect of HbA1c trajectories and other covariates to HHF recurrence risk based on the NHPP model: hazard ratios and p-values.

	Hazard Ratio (95% CI)	p-value
LowS (Base)	1	–
ModLowA	0.79 (0.6 to 1.0)	<0.01*
ModHighA	1.30 (0.5 to 2.1)	0.02*
ModLowD	1.17 (0.7 to 1.8)	0.15
ModHighD	1.89 (1.0 to 2.8)	<0.01*
ModHighV	1.94 (1.2 to 2.6)	<0.01*
HighSD	1.25 (0.8 to 1.7)	<0.01*
HighV	2.88 (2.0 to 3.7)	<0.01*
Age at diagnosis	1.02 (1.01 to 1.03)	0.99
Female (Base)	1	–
Male	1.08 (0.8 to 1.4)	0.64
Chinese (Base)	1	–
Malay	1.11 (0.7 to 1.5)	0.41
Indian	1.34 (0.8 to 1.7)	0.02*
Other races	1.17 (0.5 to 1.9)	0.05*
Baseline HbA1c	1.02 (0.9 to 1.1)	0.84
Without baseline prior complications (Base)	1	–
Established CVD	4.94 (3.5 to 6.4)	<0.01*
Prior IHD	1.42 (0.8 to 2.0)	0.03*
Prior PAD	0.99 (0.4, 1.6)	0.91
Prior HS	0.83 (0.2 to 1.5)	<0.01*
Prior IS	0.65 (0.3 to 1.0)	<0.01*
Prior TIA	0.73 (0.2 to 1.3)	<0.01*
Prior AF	2.50 (1.4 to 3.6)	<0.01*
Prior Neuropathy	1.70 (0.9 to 2.5)	<0.01*
Prior DPA	2.17 (1.1 to 3.3)	<0.01*
Prior HHF	5.16 (3.3 to 7.1)	<0.01*

*Significant covariates.

**Table 1** also summarizes the rate of type 2 diabetes medication dispensed for each class, taking into account the treatment history before the left bound of the observation window for each patient. Given that patients may receive treatment with multiple medications, the effect of these medications on the risk of HHF is complicated. Such medications can exhibit negative, neutral or positive effects (6, 7). Hence, to avoid potentially inaccurate outcomes, we opted not to directly integrate medication into the NHPP model.

### 3.4 Prediction for future HHF

Determining if a patient will develop heart failure within a specific timeframe is a key focus in clinical studies. To assess the predictive capability of the adjusted NHPP model, we explore predicting the risk of HHF in the next six months, one year, and two years following the date when patients first had complete covariates as examples. The patients are divided into a training set and a test set for analysis. Among all 194,258 subjects, only 13,099 have HHF records, accounting for 6.7%. The imbalanced case-control rate will result in the evaluation metrics not accurately reflecting the actual classification performance. For example, even if all patients are predicted to belong to the group of no HHF occurrence, the false negative rate (FNR) would remain at a low level. We adopted a down-sampling strategy to balance the case-control sample distribution. 13,099 subjects are randomly selected from the population without HHF. The newly sampled dataset consists of 26,198 patients, evenly split between those with and without HHF. 80% of subjects from both groups were randomly selected to comprise the training dataset. A total of 20,958 subjects were fed into the adjusted NHPP model for parameter estimation. The remaining 5,240 subjects (20% of the cohort) were labeled as to whether they had HHF in the next six months, one and two years after the first date they had complete covariates, respectively. The probability of having HHF for each subject in the validation set is calculated. Patients whose probability exceeds the predetermined threshold would be classified into the group with HHF.

**Figure 4:**
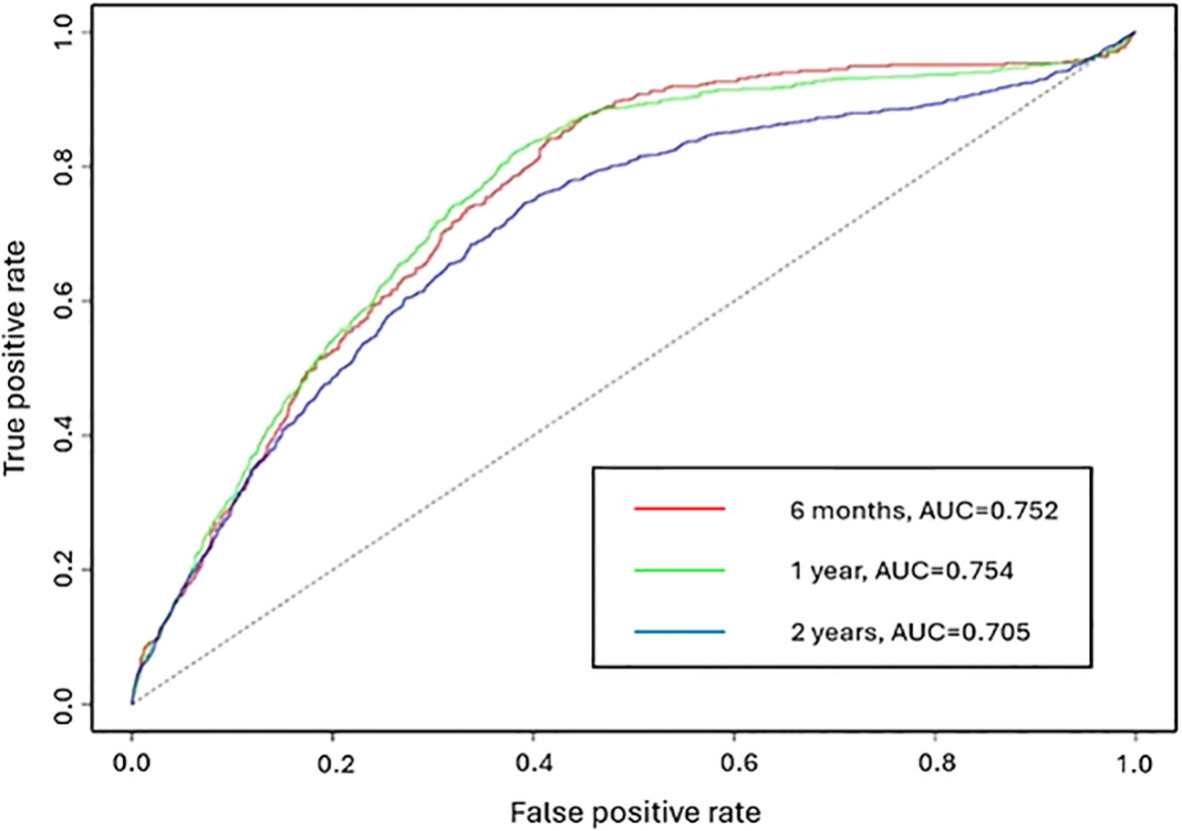
The ROC curve of the adjusted NHPP model for the prediction periods 6 months, 1 year and 2 years.

**Figure 4** displays the receiver operating characteristic (ROC) curve for 6 months, 1 year, and 2 years. AUC is the area under the ROC curve which measures the overall classification accuracy. The AUC values for the three intervals are 0.752, 0.754 and 0.705 respectively. An AUC greater than or equal to 0.7 indicates acceptable discrimination (32). The AUC values at 6 months and 1 year are similar. A lower prediction accuracy is observed for a two-year prediction period.

## 4 Discussion

This study is an extension of our previous work (24) which starts from the DDD to distinguish the latent HbA1c groups for 17,389 subjects and prove the ability of latent classes in predicting future HHF. Given the extensive cohort of 194,258 patients with type 2 diabetes and their highly unbalanced HbA1c records, the present study developed a flexible approach for distinguishing latent patient groups and tracking the progression of HbA1c levels. As a result, eight subgroups among type 2 diabetes patients were identified following similar HbA1c profiles over time. While the number of identified groups is larger than those in previous studies of individuals with newly diagnosed diabetes (21, 24, 26), the fundamental characteristics of these subgroups remained consistent, including good glycemic control (Class LowS, ModLowA and ModLowD), moderate glycemic control (Class ModHighA and ModHighD), poor glycemic control (Class ModHighV and HighV) and highly improved glycemic control (Class HighSD). The differences in the number of subgroups can be partly attributed to variations in data volume and observation periods. The utilization of natural splines accommodates greater variation in HbA1c trajectories. Subjects in Class HighD from our previous study (24) are predominately included in the newly identified Class HighV (88.7%). In addition to high HbA1c levels, Class HighV displays considerable fluctuations in glycemic control. Previous Class HighSD subjects are primarily distributed across the new Classes ModHighD, ModHighV, HighSD and HighV. While the previous Class HighSD maintained good control, the extension of the observation period from 350 weeks to 2,500 weeks in the current study reveals diverse HbA1c trajectories.

The association between the identified HbA1c trajectories and the incidence of HHF is reported in this study. Low baseline HbA1c levels may be associated with low risk of HHF, evident in both Class LowS and Class ModLowA, despite Class ModLowA having poorer glycemic control than Class LowS, which was consistent with previous studies (5, 33). Additionally, the lower rate of prior complications in Class ModLowA compared to Class LowS may contribute to its lower associated risk. However, high baseline levels are not equal to higher risk. Class HighSD has the highest baseline level followed by a sharp drop in HbA1c, reflecting effective glycemic treatment, and this trajectory is associated with a relatively low risk of HHF. In contrast, Class HighV, with high baseline values and large fluctuations, is associated with the highest risks of HHF. Class ModHighA begins with a moderate HbA1c level, and as the trend ascends in the subsequent period, the risk of HHF becomes greater compared to Class HighSD. Class ModHighD and Class ModHighA, where the former exhibits better glycemic control compared to the latter, while the latter presents a lower baseline level, demonstrate a similar risk for HHF. Thus, both baseline HbA1c levels and subsequent glycemic control may contribute to the risk of HHF in type 2 diabetes patients (34). In addition, while the previous Class HighSD has a relatively high concordance rate with newly identified Class HighSD (44.5%), it remains significantly associated with a high risk of HHF. One possible explanation could be the difference in sample size. The previous Class HighSD has a higher rate of patients with prior complications compared to the new Class HighSD, contributing to the elevated risk. Another factor to consider is the observation time. It appears that glycemic control does not have an obvious effect on the risk of HHF in the early stage, but over time, a notable reduction in incidence rates is observed.

HbA1c levels are closely associated with the antidiabetic medication usage. Classes LowS, ModLowA and ModLowD, characterized by relatively low HbA1c levels, exhibit lower rates of medication dispensation. Conversely, Classes ModHighD, ModHighV, HighSD and HighV, with higher baseline HbA1c levels, show higher medication dispensation rates. Notably, only Class HighSD demonstrates effective control and a reduced risk of HHF. This could be attributed to Class HighSD having the highest rate of SGLT2i and metformin (6), both of which have demonstrated benefits in reducing HHF risk. In contrast, Class HighSD has a similar usage rate of DPP-4 inhibitors and sulfonylureas compared to the other three classes; DPP-4 inhibitors are associated with a neutral effect, while sulfonylureas are linked to an increased HHF risk (6, 7).

Except for the HbA1c trajectories, established CVD and prior HF emerge as particularly significant contributors to an increased HHF incidence rate among the influential factors. Notably, patients with a history of prior HS, IS and TIA exhibit a lower risk of HHF compared to those without a history of stroke. These findings align with the results presented by (24), which also report a decreased risk of HHF among individuals with prior strokes, despite these factors not being statistically significant. This may be because large sample sizes are more likely to magnify the impact of covariates (35, 36). HF has been proven as a risk factor for stroke, a relationship extensively explored in studies focusing on preventing strokes in patients with HF (37). Those patients may receive treatments more targeted at stroke and HF prevention, which in turn reduces the incidence of future HHF.

The predictive ability for future events of NHPP is demonstrated. In this dataset, the status of cardiovascular complications is updated annually. The covariates incorporated in this model represent their status at the prediction start time. For the next 6 months or 1 year, where the status remains unchanged, competitive prediction accuracy is achieved. However, when predicting events 2 years into the future, the cardiovascular complications status is more likely to change during the prediction periods. Since this information is not factored into the prediction process, results in decreased prediction accuracy. This also illustrates the effectiveness of the model we proposed, emphasizing the importance of timely information for accurate predictions. Compared with our previous study (24), their prediction start time was determined by a fixed date (DDD). The present study initiates predictions when covariates information becomes available. It determines the class of new patients based on historical HbA1c values and assigns them to the class with the highest probability in the mixed effect model. The approach eliminates the constraints imposed by fixed DDD and offers a flexible and clinically relevant prediction framework.

This study further underscores the potential clinical significance of the latent subgroups revealed by LGCM. The subgroups distinguish patients by glycemic control, laying the foundation for understanding the underlying causes of variations in glycemic performance. Individuals within each identified class display corresponding HbA1c trends that reflect different levels of cardiovascular event risk. For incoming patients, it facilitates the tracking of HbA1c progression and enables predictions regarding potential HHFs given the historical trajectories and other relevant attributes. It allows early identification of high risk patients. Patients in HighV and ModHighV groups have the highest risk of recurrent HHF. Clinicians can prioritize these patients for closer monitoring and early intervention. And it supports the treatment adjustment based on trajectory trends. Patients in HighSD group showed an initially high HbA1c level followed by a rapid drop. While this group exhibited lower long-term HHF risk, abrupt declines in HbA1c could indicate aggressive medication adjustments, which might increase the risk of hypoglycemia. Clinicians may need to monitor medication titration carefully to avoid adverse effects. And it can also help personalized follow-up frequency. Patients in ModLowA group had an increased risk of HHF despite relatively low baseline HbA1c levels. This suggests a more frequent HbA1c monitoring (38).

There are several limitations of this study. First, the study does not account for the impact of medications when assessing HHF risks. When we incorporated the use of SGLT2i into the NHPP model, it showed a higher risk of HHF for patients using SGLT2i, which is contrary to known research results. This discrepancy may result from reverse causation, where patients with HF or those at higher risk for it are more likely to be prescribed SGLT2i, making it appear as though SGLT2i are associated with a higher risk of HF. To address this issue, a new model is required. Further investigation is needed to explore the relationship between glucose-lowering medications, HbA1c levels, and HHF risk. Moreover, sparse data in the later period limits the model’s ability to capture variations of HbA1c levels between 1,300 weeks and 2,500 weeks. Furthermore, the records of HHF were not available before 2007. Even though the window-observed NHPP could model the observed data accurately, the estimated risk of HHF tends to be lower than the real situation.

## 5 Conclusion

In conclusion, our findings indicate that individuals with low baseline HbA1c levels and stable HbA1c trajectories exhibit a reduced incidence of HHF compared to those with higher baseline levels. The established framework holds the potential to identify at-risk patient groups and constantly assess their risk of HHF using up-to-date medical records, even for patients with a long history of diabetes and missing HbA1c information. This is achieved through the innovative use of both LCGM with splines model and the NHPP model. This capability may empower patients with self-monitoring and risk warnings while providing clinicians with the tools to develop more personalized care strategies.

## Data Availability

The datasets presented in this article are not readily available because data cannot be shared publicly because of SingHealth Cluster Data Policy on data sharing restrictions. Data are available from the SingHealth Diabetes Registry Disease Registry Committee for researchers who meet the criteria for access to confidential data. The criteria include IRB approval, data use approval and a Research Collaboration Agreement. Requests to access the datasets should be directed to https://www.singhealth.com.sg/patient-care/specialties-services/diabetes-centre/singhealth-diabetes-registry.
