# Supplementary material for: Modeling recurrent heart failure risk in type 2 diabetes: impact of flexible HbA1c trajectories using nonhomogeneous Poisson processes
Source: Front Endocrinol (Lausanne). 2025 Apr 21;16:1472846. doi: 10.3389/fendo.2025.1472846 (PMC12051216; doi:10.3389/fendo.2025.1472846)
Supplement: Supplementary file 1 [file DataSheet1.docx]

Supplementary Material

# Supplementary Tables

**Table S1. BIC values based on different number of interior knots for two strategies with 3 latent classes.**

| Number of interior knots | | 5 | 10 | 15 | 20 | 25 | 30 | 35 | 40 |
| --- | --- | --- | --- | --- | --- | --- | --- | --- | --- |
| BIC | Equidistant | 4,021,377 | 4,003,870 | 3,991,347 | 3,981,869 | 3,975,549 | 3,970,804 | 3,967,624 | 3,965,427 |
|  | Equipotent | 3,979,647 | 3,962,139 | 3,960,622 | 3,961,295 | 3,960,340 | **3,958,899** | 3,961,231 | 3,961,069 |

**Table S2. Average BIC values for different number of classes with fixed interior knots.**

| Number of classes | 1 | 2 | 3 | 4 | 5 | 6 | 7 | 8 | 9 | 10 |
| --- | --- | --- | --- | --- | --- | --- | --- | --- | --- | --- |
| Average BIC | 606,816 | 585,019 | 572,509 | 566,601 | 564,424 | 563,215 | 562,528 | **562,415** | 562,440 | 562,555 |

**Table S3. BIC values based on different number of interior knots for two strategies with 8 latent classes.**

| Number of interior knots | | 5 | 10 | 15 | 20 | 25 | 30 | 35 | 40 |
| --- | --- | --- | --- | --- | --- | --- | --- | --- | --- |
| BIC | Equidistant | 3,445,448 | 3,442,459 | 3,441,478 | 3,438,667 | 3,440,306 | 3,465,423 | 3,461,397, | 3,458,318 |
|  | Equipotent | 3,488,769 | 3,448,616 | 3,439,406 | 3,440,668 | 3,439,769 | **3,438,436** | 3,439,878 | 3,440,406 |
